# Tick trails: the role of online recreational trail reviews in identifying risk factors and behavioral recommendations associated with tick encounters in Indiana

**DOI:** 10.1186/s12889-021-10940-4

**Published:** 2021-05-13

**Authors:** Kristina R. Anderson, Jordan Blekking, Oghenekaro Omodior

**Affiliations:** 1 Department of Health & Wellness Design, School of Public Health, Indiana University Bloomington, 1025 E. 7th Street, IN 47405 Bloomington, USA; 2 Department of Geography, College of Arts & Sciences, Indiana University Bloomington, 701 E. Kirkwood Avenue, Bloomington, IN 47405 USA

**Keywords:** Tick exposure, Risk factor, Protective behavior, Recreation, User behavior

## Abstract

**Background:**

Recreational trails abound across the United States and represent high risk areas for tick exposure. Although online reviews represent a rich source of user information, they have rarely been used in determining the risk of tick exposure during recreational trail use. Based on online user reviews and comments, the purpose of this study was to determine risk factors and behavioral recommendations associated with tick encounters (*Tick Presence*) on recreational trails in the state of Indiana, U.S.

**Methods:**

We reviewed 26,016 user comments left on AllTrails.com for 697 Indiana trails. Reviews were evaluated to determine *Tick Presence/Absence*, the total number of *Tick Presence Reviews* per trail, and multiple trail and user behavioral characteristics. We used hot spot (Getis-Ord Gi*) analysis to test the hypothesis of whether there are clusters in the number of *Tick Presence Reviews*. Pearson chi-square tests of independence evaluated whether tick presence was associated with several trail characteristics. Finally, negative binomial regression evaluated the strength of the association between the number of *Tick Presence Reviews* and several trail characteristics.

**Results:**

*Tick Presence* was recorded at 10% (*n* = 65) of trails and occurred most frequently in May. Hot spot analysis revealed statistically significant clusters of *Tick Presence Reviews* on trails in the Southern Indiana *State Region*. Results of χ^2^ tests indicated significant associations between *Tick Presence Reviews* and (a) *State Region* and (b) *Land Management Type*; Mann-Whitney U tests detected significant differences in *Tick Presence Reviews* based on *Trail Length* and *Elevation Gain.* Subsequent results of a negative binomial regression model indicated that Southern Indiana *State Region,* Federal and Private *Land Management Type*, and *Elevation Gain* were factors significantly associated with *Tick Presence Reviews.* Content of user reviews indicated several behaviors employed to prevent tick encounters, particularly *Repellent Application* and *Recreational Deterrenc*e; 25% included a behavior *Recommendation* to others.

**Conclusions:**

Online, user-generated trail reviews have the potential to serve as rich data sources for identifying recreational trails, where 1) the risk of tick exposure is great, 2) more robust active tick and tick-borne pathogen surveillance may be warranted, and 3) tailored prevention interventions are needed.

**Supplementary Information:**

The online version contains supplementary material available at 10.1186/s12889-021-10940-4.

## Background

Over the past fifteen years, reports of tick-borne diseases have grown across the United States (U.S.), with the decade from 2007 to 2017 representing a near-doubling in total reported tick-borne diseases [1, 2]. Ticks are prevalent across all 48 contiguous states, and the geographic range of tick distribution continue to expand [3, 4]. Among the various settings where exposure to zoonotic agents (tick, mosquitoes, fleas) occurs, spaces for public outdoor recreation are notable among visitors and personnel alike, and the risk associated with recreational tick encounters varies dramatically based on tick abundance as well as temporal and spatial factors [5–9].

In one survey of state parks across Oklahoma, researchers identified that the potential tick encounter rate varied from 32.1 ticks per hour at one park in May to less than one per hour at another park during the peak summer season [6]. The role of public recreational sites as sites of tick exposure can also vary by U.S. region. In Connecticut, where ticks are largely endemic, time spent in one’s old yard, as opposed to public recreation sites, was significantly more predictive of individuals’ tick detection [10]. Conversely, in a study of tick exposure among California residents, tick encounters occurred in natural areas of recreation more than 80% of the time, in contrast to peri-domestic settings (≤15%) [11]. Still, few inquiries into recreational tick exposures have taken place in the U.S.’s Midwest region. Additionally, extant literature has identified limited characteristics associated with tick presence in recreational spaces, such as specific habitat attributes [12, 13]. For instance, whereas evidence indicates that a property’s land management level influences its scope of tick surveillance and pathogen detection initiatives [14], the association between various management levels and tick presence is less well understood.

The body of literature examining behaviors associated with known or anticipated tick encounters at sites of outdoor recreation is also limited; one study conducted in Florida found that less than one-third of visitors acknowledged practicing tick protective behaviors, such as applying insect repellent, each time they visit a recreational site, despite the majority indicating high intention to engage in those behaviors [15]. Other research has evaluated the role of recreational deterrence, wherein an individual avoids outdoor recreation in a given location due to anticipated or known tick presence, although the rate at which individuals adopt this strategy ranges widely, from 7 to 59% [16–18].

Public outdoor recreation spaces also represent a particularly unique challenge from an epidemiological, public health perspective: Because users of these spaces often include nonresidents of the local area who may recreate in one area and travel prior to discovering a tick bite and/or seeking medical treatment due to tick-borne disease symptoms, discrepancies can occur in understanding local risks of vector-borne disease [19]. Additionally, even if the specific site of vector exposure is identified, the degree to which this knowledge is successfully communicated to the relevant, local public health agency is unknown [19]. Consequently, these challenges represent a need for innovative approaches to identifying and understanding tick encounters in areas of public outdoor recreation.

To better identify and understand recreational tick encounters, this study seeks to employ online user reviews of recreational trails in Indiana, U.S. to address three gaps in extant literature. Our first research objective seeks to use online user reviews to pinpoint locations of tick encounter (*Tick Presence)*, as well as identify specific spatial and temporal patterns in *Tick Presence*. Leading scholars have denoted the need for this type of scholarship to improve the use of spatial epidemiologic data in understanding vector-borne diseases, particularly calling for identification of patterns at finer scales, i.e., more detailed than county-level [14, 20]. Second, given available data with each trail’s online profile, we seek to identify trail characteristics, such as *Land Management Type,* that are associated with *The Presence*. Our final research objective seeks to understand behavioral responses associated with *Tick Presence* at public sites of outdoor recreation, a noteworthy contribution, as existing research has generally relied upon survey-based, self-reported practices, a strategy which depends on recall and often results in over-reporting of socially-desired behaviors [21]. Concomitantly, given that current determinations of *Tick Presence* generally occur through active surveillance processes, e.g. deployment of field-based teams to conduct in-situ drag sampling [6, 7, 12] we also contribute a methodological advancement which is cheaper than population-based surveys and can be employed even with limited resources through analysis of online user reviews.

Our study findings and methodological innovation may have important implications for 1) developing tailored and targeted population-level public health interventions aimed at tick exposure risk reduction in Indiana, 2) identifying novel approaches in the collection of data for passive surveillance, not just for tick exposure risk, but for other vector-borne disease risks, and 3) initiating similar studies not only across every U.S. state, but internationally [22].

## Methods

### Data source and collection

AllTrails was employed as the online user review data content source due to its prominence within the recreational trail user community; as of October 2020, the website reported global trail use on more than 100,000 trails by greater than 20 million “explorers” [22]. AllTrails allows users to search for trails by state, city, park site, and average user rating, among other characteristics. Each individual trail page reports on several trail characteristics, such as elevation gain; lists user reviews; and provides a trail map. Users are encouraged to rate trails and leave reviews, which can include photos and/or waypoints to demark a specific feature along the trail. Users may access limited information via free web access or pay a monthly subscription fee for premium access.

Every trail located in [1] the state of Indiana and [2] included in the AllTrails.com database as of October 31, 2020 was included in the study. This resulted in a review of 697 trails across Indiana. For each trail, additional data was recorded including longitude/latitude of the trailhead, *Trail length* (in miles), *Elevation Gain* (in feet), *Route Type*, and associated park/nature site/location, as indicated by AllTrails.com. The review date was also recorded as a proxy for exposure date, under the assumption that the review was posted promptly following the trail visit the same day, or very shortly thereafter. Based on spatial location denoted by longitude/latitude, trails were coded for presence in the north, central, and southern regions of the state according to County Surveyors Association of Indiana [23] designation, resulting in a *State Region* variable. Additionally, the *Land Management Type* (e.g. municipal, county, state, federal, private, and unknown) of each was recorded; this was derived from either the indicated park/nature site name (e.g. “Brown County State Park”, indicating state ownership), or an internet search, if the ownership type was not initially apparent.

### Inclusion/exclusion criteria

All reviews recorded during an approximately 3-season period from April 1–October 31st over a two-year period (2019 and 2020 seasons) were evaluated for a reference to “tick” or “ticks”, excluding terms such as “stick” or “sticker” that are not relevant in meaning. These dates were selected because, April through October reflects the general period of most tick activity in the Midwestern US [24] as well as general recreational use of trails in Indiana [25].

### Data analysis

After a search to identify reviews including the term “tick(s)”, all tick-related reviews were recorded. The unit of analysis reflected each singular AllTrails user review, and one member of the research team initially evaluated each review for content broadly related to tick presence/absence, tick description (e.g. size, count), protective behaviors, and tick-related recreational deterrence. These themes were based on a cursory review of tick-related reviews from 20 Indiana trails, a priori content area knowledge of the research team, as well as the example of Liu, Pennington-Gray [26] who adopted a similar methodology in their study of user bed bug perceptions on TripAdvisor.com. After completing this initial evaluation, the researchers met to discuss these initial findings, finalize codes and group them by theme; during this time the research team also collectively interpreted 19 reviews that had been flagged by the initial researcher who was uncertain whether those reviews reflected a general comment about ticks or an actual tick encounter. After this meeting, one researcher proceeded to reassess the reviews again in line with the revised codes and definitions.

The final themes included tick experience (*Tick Presence*, *Tick Absence,* and *Tick General*), tick protective behaviors (including three self-reported behaviors including *Repellent Application*, *Protective Clothing*, and *Shower Post-Recreation* as well as one reflecting *Recommendation* of protective behavior(s) to other users/readers, as well as a theme related to *Recreational Deterrence*. A complete list and definition of each is provided in Table 1.

**Table 1:** Tick-Related User Review Themes (*N* = 147)

Theme	Concept	Definition	n, %
Tick experience			
	Tick presence	User explicitly indicates presence of tick(s) in review	124 (84.4%)
	Tick absence	User explicitly indicates absence of tick(s) in review	20 (13.6%)
	Tick general	User makes a general comment about tick(s), but does not clearly indicate tick encounter in review	3 (2.04%)
Tick protective behavior			
	Repellent application	Reference to having applied insect repellent prior to tick encounter	14 (9.52%)
	Protective clothing	Reference to having made intentional clothing choices (e.g. long clothes, light-colored clothes, or treated clothes) prior to tick encounter	5 (3.40%)
	Shower post-recreation	Reference to having taken a shower post recreational activity	2 (1.36%)
	Recommendation	Review makes explicit recommendation to other users, which includes one or more suggestions for how to prevent tick bites	37 (25.17%)
Recreational deterrence			
	Recreational deterrence	Review suggests that the user disengaged from use of that trail in the moment or plans to avoid the trail in the future due to the tick encounter	11 (7.48%)

Beyond evaluation of user review narrative content, quantitative analysis also evaluated *Tick Presence* as a binary per trail, wherein if any review of that trail indicated *Tick Presence,* the value would be set at 1 (no reviews indicating tick presence at a given trail resulted in 0). A total number of reviews indicating tick presence per trail was also calculated as *Tick Presence Reviews;* additionally, the percentage of each’s trail total reviews indicating tick presence was also calculated (i.e. *Tick Presence Reviews % of Total)*. Maps reflecting these total and relative counts of *Tick Presence* were created using Tableau 2020.2. Hot spot (Getis-Ord Gi*) analysis was conducted in ArcGIS Desktop 10.7 to identify significant spatial clusters of trails within Indiana. χ2 tests of association evaluated relationships between *Tick Presence* and several trail characteristic variables including *State Region, Land Management Type,* and *Route Type;* Mann-Whitney U tests were employed to evaluate differences in *Trail Length* and *Elevation Gain* based on *Tick Presence*, given that these variables did not reflect normal distributions.

We then utilized a negative binomial regression to evaluate the relationship between these variables and the outcome variable of *Tick Presence Reviews,* a value which reflected a count of all comments indicating *Tick Presence* at each trail. While Poisson models are frequently used to model count data, our data exhibited over-dispersion (wherein conditional variances exceeded that of conditional means), thus calling for the use of the negative binomial model. Statistical analysis was conducted with IBM© SPSS version 27.0 (https://www.ibm.com/products/spss-statistics).

## Results

Over the two-year period, users recorded 26,016 trail reviews: 6729 in 2019 and 19,287 in 2020. Of these, 147 (0.57%) reviews referenced ticks; 38 in 2019 and 109 in 2020. However, not all of these were indicative of a tick encounter. Of the 147 reviews referencing ticks, 124 indicated *Tick Presence,* 20 indicated *Tick Absence and* 3 indicated *Tick General. Tick Presence* was recorded at 65 Indiana trails (of 697 trails total), or 9.46% of trails.

A sample of those 65 trails, illustrating rank order of the top 10 trails by (a) tick-related reviews as a percentage of total reviews and (b) total number of tick-related reviews are outlined in Table 2. A full summary of the 65 trails is provided in Appendix A. Notably, most (*n* = 43) of those trails indicated tick encounter by just one user review. The remaining 22 indicated tick presence by more than one user review; the maximum number of tick-related reviews of any trail was 13 (Two Lakes Loop Trail, Branchville, IN).

**Table 2:** Tick Presence Reviews at Select Trails

No.	Trail	Tick Presence Reviews			All User Reviews			Tick Presence Reviews % of Total
		2019	2020	Total	2019	2020	Total	
Top 10 Trails by Tick Presence as Percentage of Total								
1	Rabbit Hash Trail	1	3	4	1	4	5	80%
2	Columbia Mine Preserve Loop	0	4	4	1	10	11	36%
3	German Ridge Lake Trail	0	3	3	3	10	13	23%
4	Birdseye Trail	1	2	3	6	12	18	17%
4	Trail 8 and Pine Bluff Shelter	0	2	2	2	10	12	17%
4	Thomas Ciurus Nature Preserve	1	0	1	2	4	6	17%
7	Violet and Louis Calli Nature Preserve	0	3	3	3	18	21	14%
7	Shaw Lake Loop Trail	0	2	2	5	9	14	14%
7	Ropchan Memorial Trail	0	1	1	1	6	7	14%
8	Two Lakes Loop Trail	6	7	13	35	68	103	13%
8	Turkey Roost Run Trail	1	1	2	7	9	16	13%
Top 10 Trails by Total Tick Presence								
1	Two Lakes Loop Trail	6	7	13	35	68	103	13%
2	Adventure Hiking Trail	1	5	6	25	85	110	5%
3	Laura Hare Nature Preserve Trail at Downey Hill Full Loop	1	4	5	27	104	131	4%
4	Lake Monroe Peninsula Trail	2	2	4	74	137	211	2%
4	Patoka Lake Main Trail	0	4	4	17	49	66	6%
4	Shawnee and Lenape Trail Loop	0	4	4	6	35	41	10%
4	Columbia Mine Preserve Loop	0	4	4	1	10	11	36%
4	Rabbit Hash Trail	1	3	4	1	4	5	80%
9	Cowles Bog Trail	1	2	3	114	305	419	1%
9	Sycamore Loop Trail	1	2	3	38	75	113	3%
9	Tipsaw Lake Trail	1	2	3	8	27	35	9%
9	Allen’s Creek	1	2	3	3	29	32	9%
9	Hickory Ridge Trail	0	3	3	9	20	29	10%
9	Cave River Valley Trail	0	3	3	3	26	29	10%
9	Violet and Louis Calli Nature Preserve	0	3	3	3	18	21	14%
9	Birdseye Trail	1	2	3	6	12	18	17%
9	German Ridge Lake Trail	0	3	3	3	10	13	23%

### Spatial and temporal tick encounter patterns

**Fig. 1:**
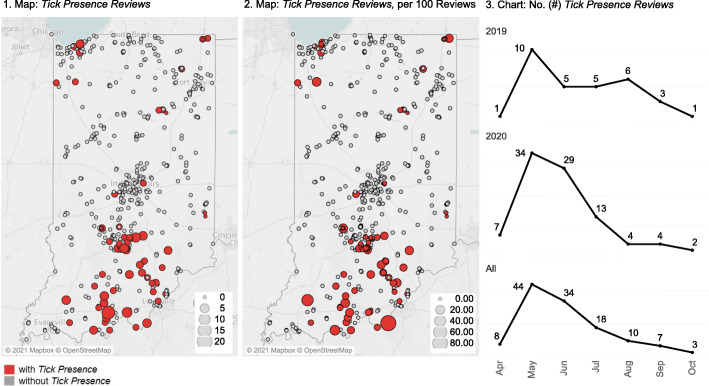
Tick Encounter Patterns, Tick Presence Reviews = 124, all Indiana trails *N* = 697

**Fig. 2:**
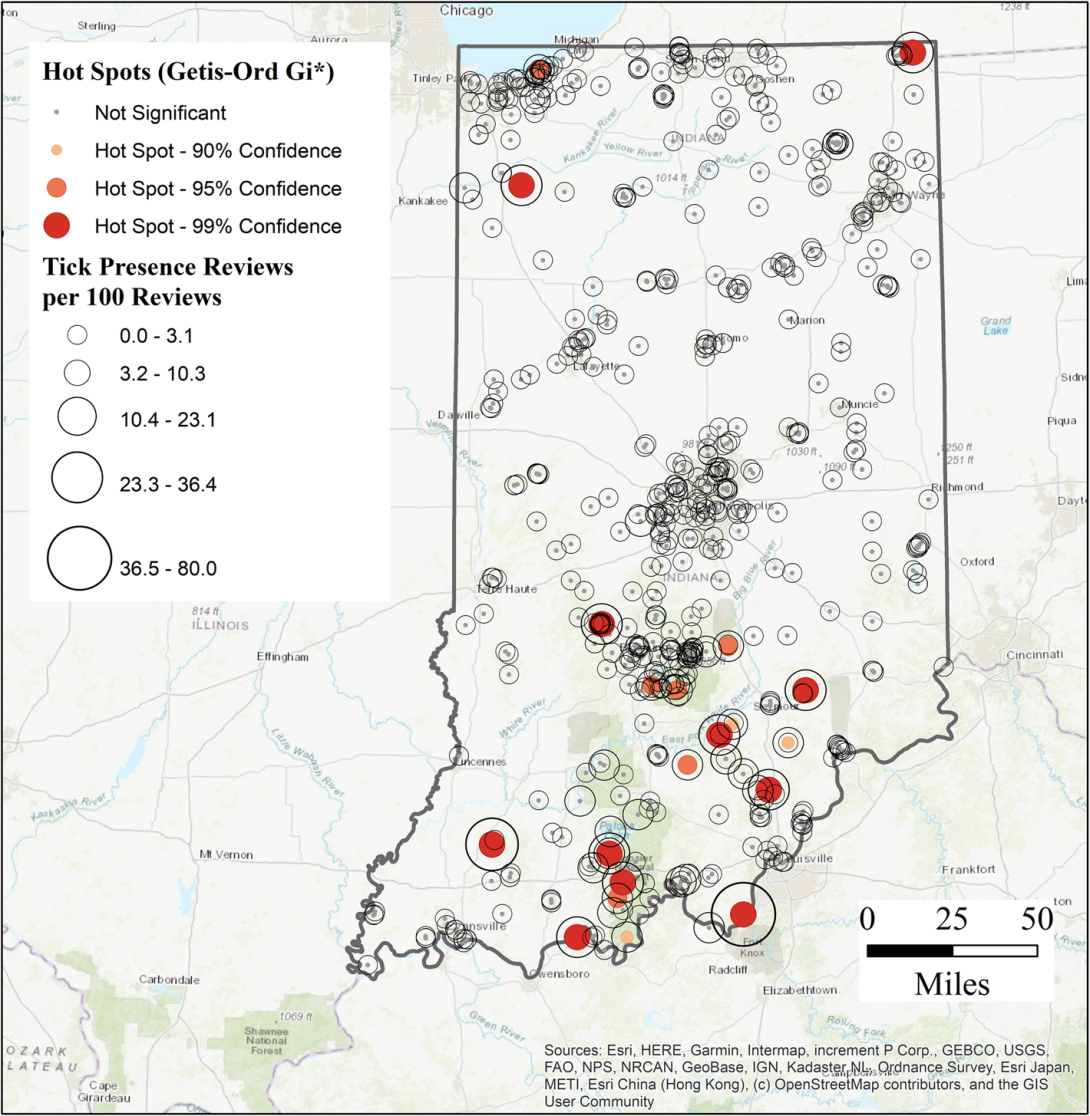
Hot spot Analysis of Tick presence Reviews per 100, all Indiana trails *N* = 697

Figure 1 illustrates all 697 trails across the Hoosier state, with and without *Tick Presence*, as well as total *Tick Presence Reviews*, as shown through graduated symbol size. Visually, the Southern Indiana region reflects higher rates of reported tick encounters, particularly near the Hoosier National Forest (adjacent to the Ohio River). Subsequent hot spot analysis further identified a collection of hot spot values in this area (Fig. 2). No cold spots were identified in the analysis.

In evaluating seasonality of tick encounters, the month in which most tick encounters (*Tick Presence Reviews)* were reported was May, with steady decline as the summer proceeded into the fall season, with October representing the month indicating the fewest reported tick encounters (Fig. 1). This pattern was generally reflected both in 2019 and 2020, as well as overall.

### Trail characteristic associations

Analysis of trail characteristic associations (Table 3) indicated significant associations between *Tick Presence* and trails’ *State Region* χ2 (2, *N* = 697) = 55.30, *p* <  0.001; notably, 77% (*n* = 50) of trails indicating tick presence were located in the Southern portion of the state, whereas only 31% (*n* = 198) of trails that did not indicate tick presence were located in this region. Significant associations were also identified between *Tick Presence* and *Land Management Type* χ2 (5, *N* = 697) = 61.15, *p* <  0.001. The plurality of trails reflected state land management (36% overall, *n* = 251) although this varied by tick presence (48%, *n* = 31) and the lack thereof (35%, *n* = 220). Loop was the most frequent trail route type (74%, *n* = 516); however, no significant associations were identified based on *Route Type* and *Tick Presence* χ2 (2, N = 697) = 2.11, *p* = 0.348. While the average *Trail Length* was 4.4 miles (Mdn = 2.4), this metric was significantly higher on trails indicating *Tick Presence* (M = 6.2, Mdn = 4.4) than those that did not (M = 4.2, Mdn = 2.2) (*U =* 12,552, *p* <  0.001). Similarly, the average *Elevation Gain* on trails was 320.2 miles (Mdn = 124), with a significantly higher average gain on trails indicating *Tick Presence* (M = 721.3, Mdn = 472) than those that did not (M = 278.9, Mdn = 110) (*U = 9460*, *p* <  0.001).

**Table 3:** Description of Indiana Trails by Trail Characteristics and Tick Presence (N = 697)

	Trails indicating Tick Presence		Trails not indicating *Tick Presence*		Overall		*p*-value
	n	%	n	%	n	%	
Count	65	–	632	–	697	100%	–
State Region							
North	12	18%	222	35%	234	34%	< 0.001***
Central	3	5%	212	34%	215	31%	
South	50	77%	198	31%	248	36%	
Land Management Type							
Municipal	3	5%	133	21%	136	20%	< 0.001***
County	1	2%	78	12%	79	11%	
State	31	48%	220	35%	251	36%	
Federal	22	34%	48	8%	70	10%	
Private	6	9%	101	16%	107	15%	
Unknown	2	3%	52	8%	54	8%	
Route Type							
Loop	53	82%	463	73%	516	74%	0.348
Out & back	9	14%	129	20%	138	20%	
Point to point	3	5%	40	6%	43	6%	
Average Trail Length, mi	6.2	–	4.2	–	4.4	–	< 0.001***
(Median, mi)	4.4		2.2		2.4		
Average Elevation Gain, ft	721.3	–	278.9	–	319.9	–	< 0.001***
(Median, ft)	472		111		124		

Last column reflects results of Chi-square tests of independence for State Region, Land Management Type, and Route Type variables. Last column reflects results of Mann–Whitney U nonparametric tests of differences for Average trail length (mi) and Average elevation gain (ft) variables

*p* < 0.05; **p < 0.01, ***p < 0.001

### Negative binomial model results

The negative binomial regression model identified several statistically significant associations between tick trail characteristics and *Tick Presence Reviews* (Table 4). Holding all other variables in the model constant and compared to trails located in the Northern *State Region*, trails located in the Southern *State Region* were significantly more associated with each unit increase in *Tick Presence Reviews* (*p* <  0.001). Additionally, trails reflecting Federal (*p* <  0.001) and Private *Land Management Type* (*p* <  0.047) were significantly more likely than trails of Municipal land management to be associated with each unit increase in *Tick Presence Reviews,* ceteris paribus. Finally, after holding all other model variables constant, *Elevation Gain* (in feet) was also found to be significantly associated with each unit increase in *Tick Presence Reviews (p* = 0.050).

**Table 4:** Negative Binomial Regression of Tick Presence Reviews (*N* = 697)

	Response Variable:		
	*Tick Presence Reviews*		
	Estimate	Std. Error	*p*
State Region			
North (Reference)	0.000		
Central	−0.923	0.618	0.135
South	1.295	0.379	0.001***
Land Management Type			
Municipal (Reference)	0.000		
County	−0.751	1.219	0.538
State	1.231	0.680	0.070
Federal	2.415	0.710	0.001***
Private	1.431	0.721	0.047*
Other	1.170	0.875	0.181
Route Type			
Loop (Reference)	0.000		
Out & back	−0.127	0.401	0.751
Point to point	−1.232	0.878	0.161
Trail Length (in mi)	−0.095	0.071	0.180
Elevation Gain(in feet)	0.001	0.001	0.050*

* indicates *p <* 0.05*;* ** indicates *p* < 0.01; *** indicates *p* < 0.001

### Protective behaviors

Beyond general comments indicating whether the user encountered a tick(s) or not, one recurrent theme identified across several reviews reflected the use and recommendation of protective behaviors known to either repel ticks or prevent tick bites. Several users explicitly indicated that they had engaged in *Repellent Application* (*n* = 14/147, 9.52%) during their recreational trail use; notably, of these, 11 had noted *Tick Presence* while on the trail and 4 had noted *Tick Absence*. Fewer users reported other protective behaviors, including wearing *Protective Clothing* that they perceived would aide in preventing a tick bite—e.g., wearing pants and long socks or wearing light color clothing (*n* = 5/147, 3.40%). Only two had indicated that they took a *Shower Post-Recreation* and used that activity to search for tick bites.

A larger proportion of tick-related reviews included a tick protective behavior *Recommendation,* wherein they suggest to fellow website users that they practice a protective behavior at this trail (*n* = 37/147, 25.17%).

### Recreational deterrence

Another recurrent theme was that of *Recreational Deterrence*. This portion of tick-related reviews (11/147, 7.48%) included some reference to current or future deterrence from recreation at the specific trail of the review. More than half of these reviews [6] indicated that the ticks deterred recreation in the moment, e.g. the user turned around or shortened their hike due to the tick(s) encountered. The others [5] indicating that they would avoid the trail in the future, often in the context of avoiding that location during a particular season or month.

## Discussion

A persistent challenge associated with reducing tick-borne illnesses is the difficulty in determining the location of actual human-tick encounters [27]. A strength of the present study is that it provides evidence of such encounters, while many other surveillance methods, such as drag sampling or CO_2_-baited tick traps, do not. [28] By analyzing data obtained from online, user-generated trail reviews, we (a) pinpointed specific trails of reported *Tick Presence*, (b) determined spatial and temporal patterns of *Tick Presence*, (c) identified the trail characteristics that are significantly associated with *Tick Presence Reviews*, and (d) explored the role of tick encounter in trail users’ adaptive behaviors or recreational deterrence.

Our results indicated that online user reviews showed *Tick Presence* in all three regions of the state, although *Tick Presence* occurred significantly more frequently in the Hoosier state’s southern region, an area characterized by dense plots of forested, public lands, particularly the Hoosier National Forest, in contrast to central and northern portions of the state [29]. This finding supports other research which has identified an association between the state’s southern region and increased tick presence, particularly by specific species (*Amblyomma americanum*) and settings (peri-domestic) [30, 31], as well as tick-borne disease diagnoses [32]. Given this body of evidence, systematic active surveillance of tick populations, as well as coordinated public health efforts regarding tick borne disease awareness and prevention, might well be targeted to recreational trails in this region. Online user reviews also indicate a peak in *Tick Presence* in May, and a tapering in reported *Tick Presence* through the subsequent summer and fall months. This observed pattern is generally in agreement with active tick surveillance and recreational trail use patterns in Indiana, which have indicated that tick activity and recreational trail use are highest during late spring to early summer [24, 33]; consequently, communication efforts to inform trail users of increased, seasonal late Spring risks may be warranted.

Additionally, results revealed that federal *Land Management* and a trail’s *Elevation Gain* both were significantly associated with the number of *Tick Presence Reviews*. Given that others have indicated that sub-state level (municipal, county) governmental entities may need increased support for tick and pathogen surveillance [14], the association with *Tick Presence Reviews* identified here instead reinforces the crucial role of federal land management agencies in tick surveillance and control practices. However, the association between private land management—frequently under the control of a local land trust—and *Tick Presence Reviews* warrants further investigation. While not “public” they are generally “publicly accessible” and may represent an opportunity in tick surveillance efforts and research, as land trusts can aide in mitigating environmental problems [34]. The significant association between *Elevation Gain* and *Tick Presence Reviews* is also notable; we perceive that this trail characteristic may serve as a surrogate for the trail’s terrain and ruggedness*.* As such, *Elevation Gain* may serve as a proxy indicator of users’ travel to higher-risk, undeveloped and more remote habitats, increasing the odds of tick encounter [8]; this finding warrants increased efforts to recommend PPB adoption among users who recreate on more arduous trails.

Qualitative data reflecting the adoption and recommendation of protective behaviors, as well as *Recreational Deterrence,* illuminated important context to the tick-related user experience. *Repellent Application* was the most frequently reported tick protective behavior, a finding that is aligned with extant literature which often places this behavior amongst the top tick protective behaviors generally [35, 36] and in Indiana, specifically [37, 38]. Other behaviors (*Protective Clothing, Shower Post-Recreation)* were mentioned in fewer than 5% of tick-related user reviews. *Recreational Deterrence* was also mentioned in a subset of reviews, indicating the degree to which a tick(s) encounter can negatively impact current and future outdoor recreation experiences. However, given the open-ended nature of a user review, it is feasible that users practiced a tick protective behavior or were deterred but omitted those details, especially with respect to an activity that occurs post-exposure, usually in the privacy of one’s own home (e.g. *Shower Post-Recreation)*. To that end, one key purpose of an online user review is often to share important information with other, future users; this may explain why, of the themes evaluated, behavioral *Recommendation,* wherein a user recommends that other users practice a protective behavior(s), appeared most frequently.

One limitation to the generalizability of this study’s findings is that tick abundance can vary dramatically along difference sections of an individual trail, given changes in ecological conditions (e.g. areas untreated by a controlled burn) and land use characteristics (e.g. pasture land) [12, 13]; this level of data granularity was not captured in our data. Additionally, the methods applied likely underreport *Tick Presence* by users on online trail review social media websites, particularly because AllTrail.com users were not aware that their review(s) would be analyzed for this content; as a result, it is possible that users encountered ticks but made no such indication in their online review. Additionally, there would be users who encountered ticks but were not AllTrail.com users or those who do use AllTrails.com, but do not leave trail reviews. It is also possible that a recreational trail user may have acquired a tick elsewhere, incorrectly assuming the exposure occurred at the trail reviewed. Still, we believe our methods reflect minimal recall bias, instead providing generally unbiased testament of recreational trail users’ tick encounters.

## Conclusions

We believe this research represents a low-cost and promising tool for identifying specific recreational trails that warrant more robust active surveillance, particularly at locations that may be under-surveilled, like municipal and county locations [14] as well as those managed by private institutions, such as local land trusts. Furthermore, despite the online and voluntary nature of data collected, identification of ticks at nearly 10% of trails can be contextualized within comparable research; in a study examining presence of *Ixodes scapulari* along more than 900 km of the Appalachian trail from West Virginia to Vermont, ticks presence was indicated at 21 (4.3%) of actively surveilled sites (*N* = 489) [39]. Finally, research of this kind may also be particularly useful given its ability to summarize or improve peer-to-peer communication; individuals who are fearful of ticks and seek to avoid areas of documented tick encounters, such as those peoples who experience chronic symptoms following Lyme Disease, may use the data captured here to avoid specific trails [40]. Our results also indicate that trail review websites may want to broaden the categories of variables collected and reported; for instance, a binary yes/no indicator of *Tick Presence* may inform future recreational trail/website users.

Tick-borne diseases represent the most common vector-borne diseases reported nationwide and have more than doubled over the past two decades [41]. Still, with this study we do not seek to discourage outdoor recreation, with its many associated positive health outcomes [42, 43], but instead seek to identify recreational trail sites of known tick encounters as one part of the process in preventing vector-borne disease [44]. Given the difficulty in controlling and preventing vector-borne diseases, we believe that analysis of online, user-generated reviews represents an innovative method in identifying the patterns of activities and behaviors that increase tick exposure. Going forward, future research might seek to (a) validate tick populations identified here through active surveillance or (b) evaluate the transferability of our methods to other regions, e.g. parts of New England, where many tick-borne diseases are endemic, or the Mountain West, where land is predominantly publicly owned.

## Supplementary Information

**Additional file 1.**

## Data Availability

The dataset reflecting *Tick Presence Review* on a per-trail basis are included in this article’s supplementary information files. The underlying data reflecting verbatim user review comments are not available due the identifiable nature of this qualitative data, as their content can be matched to individual user accounts publicly accessible online.
