# Supplementary material for: Tick trails: the role of online recreational trail reviews in identifying risk factors and behavioral recommendations associated with tick encounters in Indiana
Source: BMC Public Health. 2021 May 13;21:908. doi: 10.1186/s12889-021-10940-4 (PMC8117608; doi:10.1186/s12889-021-10940-4)
Supplement: Supplementary file 1 — Additional file 1. [file 12889_2021_10940_MOESM1_ESM.docx]

*Trails indicating Tick Encounter by Tick Presence as % of Total User Reviews (n = 65)*

| No. | Trail | Location | User Reviews | | | Tick Presence | | | Tick Presence % of Total |
| --- | --- | --- | --- | --- | --- | --- | --- | --- | --- |
|  |  |  | 2019 | 2020 | Total | 2019 | 2020 | Total |  |
| 1 | Rabbit Hash Trail | Elizabeth, IN | 1 | 4 | 5 | 1 | 3 | 4 | 80% |
| 2 | Columbia Mine Preserve Loop | Patoka River National Wildlife Refuge | 1 | 10 | 11 | 0 | 4 | 4 | 36% |
| 3 | German Ridge Lake Trail | Rome, IN | 3 | 10 | 13 | 0 | 3 | 3 | 23% |
| 4 | Birdseye Trail | Birdseye, IN | 6 | 12 | 18 | 1 | 2 | 3 | 17% |
| 5 | Trail 8 and Pine Bluff Shelter | McCormicks Creek State Park | 2 | 10 | 12 | 0 | 2 | 2 | 17% |
| 6 | Thomas Ciurus Nature Preserve | Demotte, Indiana | 2 | 4 | 6 | 1 | 0 | 1 | 17% |
| 7 | Violet and Louis Calli Nature Preserve | North Vernon, Indiana | 3 | 18 | 21 | 0 | 3 | 3 | 14% |
| 8 | Shaw Lake Loop Trail | Clark State Forest | 5 | 9 | 14 | 0 | 2 | 2 | 14% |
| 9 | Ropchan Memorial Trail | Fremont, IN | 1 | 6 | 7 | 0 | 1 | 1 | 14% |
| 10 | Two Lakes Loop Trail | Branchville, Indiana | 35 | 68 | 103 | 6 | 7 | 13 | 13% |
| 11 | Turkey Roost Run Trail | Jackson Washington State Forest | 7 | 9 | 16 | 1 | 1 | 2 | 13% |
| 12 | Hickory Ridge Trail | Norman, Indiana | 9 | 20 | 29 | 0 | 3 | 3 | 10% |
| 13 | Cave River Valley Trail | Cave River Valley Natural Area | 3 | 26 | 29 | 0 | 3 | 3 | 10% |
| 14 | Shawnee and Lenape Trail Loop | Sycamore Land Trust | 6 | 35 | 41 | 0 | 4 | 4 | 10% |
| 15 | Allen's Creek | Allens Creek State Park | 3 | 29 | 32 | 1 | 2 | 3 | 9% |
| 16 | #410 Trail 3 | Indiana Dunes National Park | 5 | 6 | 11 | 0 | 1 | 1 | 9% |
| 17 | Birdseye Trail Full Loop | Birdseye, IN | 3 | 8 | 11 | 0 | 1 | 1 | 9% |
| 18 | Tipsaw Lake Trail | Leopold, IN | 8 | 27 | 35 | 1 | 2 | 3 | 9% |
| 19 | German Ridge Trail | German Ridge Recreation Area | 1 | 11 | 12 | 1 | 0 | 1 | 8% |
| 20 | Hardy Lake Island, Outward Bound, and Cemetary Trail Loop | Scottsburg, Indiana | 0 | 12 | 12 | 0 | 1 | 1 | 8% |
| 21 | Sawmill Hollow Trail | Brownstown, IN | 1 | 12 | 13 | 0 | 1 | 1 | 8% |
| 22 | Axsom Branch Loop | Charles C. Deam Wilderness | 3 | 11 | 14 | 0 | 1 | 1 | 7% |
| 23 | Meadowbrook Nature Preserve Loop | Meadowbrook Nature Preserve | 6 | 22 | 28 | 0 | 2 | 2 | 7% |
| 24 | Saddle Lake Loop | Tell City, Indiana | 2 | 13 | 15 | 1 | 0 | 1 | 7% |
| 25 | Trail 3 to Trail 12 Loop | Chain O' Lakes State Park | 7 | 8 | 15 | 0 | 1 | 1 | 7% |
| 26 | Knobstone Trail Loop from Spurgeon Hollow Trailhead | Jackson Washington State Forest | 3 | 12 | 15 | 0 | 1 | 1 | 7% |
| 27 | Twin Bridges Loop Trail | Danville, IN | 3 | 12 | 15 | 0 | 1 | 1 | 7% |
| 28 | Patoka Lake Main Trail | Newtown-Stewart State Recreation Area | 17 | 49 | 66 | 0 | 4 | 4 | 6% |
| 29 | Knobstone Trail: New Chapel to Leota | Clark State Forest | 8 | 10 | 18 | 0 | 1 | 1 | 6% |
| 30 | Knobstone Trail: Elk Creek to Spurgeon Hollow | Salem, IN | 8 | 10 | 18 | 1 | 0 | 1 | 6% |
| 31 | Adventure Hiking Trail | O'Bannon Woods State Park | 25 | 85 | 110 | 1 | 5 | 6 | 5% |
| 32 | Knobstone Trail: Pixley Knob to Jackson Rd Trailhead | Clark State Forest | 7 | 12 | 19 | 1 | 0 | 1 | 5% |
| 33 | O'Bannon Woods Trail | O'Bannon Woods State Park | 11 | 10 | 21 | 0 | 1 | 1 | 5% |
| 34 | LaSalle Fish and Wildlife Aea Loop | Lasalle Fish and Wildlife Area | 5 | 16 | 21 | 0 | 1 | 1 | 5% |
| 35 | German Ridge North Loop via Gerald Road | German Ridge Recreation Area | 10 | 11 | 21 | 1 | 0 | 1 | 5% |
| 36 | Laura Hare Nature Preserve Trail at Downey Hill Full Loop | Laura Hare Nature Preserve at Downey Hill | 27 | 104 | 131 | 1 | 4 | 5 | 4% |
| 37 | Tank Spring Trail | Hoosier National Forest | 0 | 28 | 28 | 0 | 1 | 1 | 4% |
| 38 | Spring Valley Trail | Springs Valley Recreation Area | 9 | 20 | 29 | 0 | 1 | 1 | 3% |
| 39 | Knobstone Trail: Spurgeon Hollow to Delaney Park | Jackson Washington State Forest | 22 | 37 | 59 | 0 | 2 | 2 | 3% |
| 40 | Ferdinand State Forest | Ferdinand State Forest | 7 | 23 | 30 | 0 | 1 | 1 | 3% |
| 41 | Knob Lake and Pinnacle Loop Trail | Jackson Washington State Forest | 10 | 22 | 32 | 0 | 1 | 1 | 3% |
| 42 | Sycamore Loop Trail | Charles C. Deam Widlenress | 38 | 75 | 113 | 1 | 2 | 3 | 3% |
| 43 | Scales Mountain Bike Trail | Scales Lake County Park | 10 | 33 | 43 | 1 | 0 | 1 | 2% |
| 44 | Hayes Trail and Grubb Ridge Loop | Charles C. Deam Wilderness | 5 | 40 | 45 | 0 | 1 | 1 | 2% |
| 45 | Salamonie River State Forest Park Loop | Salamonie River State Forest | 14 | 38 | 52 | 0 | 1 | 1 | 2% |
| 46 | Lake Monroe Peninsula Trail | Charles C. Deam Wilderness | 74 | 137 | 211 | 2 | 2 | 4 | 2% |
| 47 | Aynes Loop | Brown County State Park | 10 | 45 | 55 | 0 | 1 | 1 | 2% |
| 48 | Knobstone Trail: Deam Lake to Bartle Knob Road | Deam Lake State Recreation Area | 19 | 37 | 56 | 1 | 0 | 1 | 2% |
| 49 | Charlestown Trail #5 | Charlestown State Park | 10 | 47 | 57 | 1 | 0 | 1 | 2% |
| 50 | Hathaway Nature Preserve Loop | Hathaway Nature Preserve | 14 | 46 | 60 | 1 | 0 | 1 | 2% |
| 51 | Flatwoods Trail | Lapping Park | 13 | 49 | 62 | 0 | 1 | 1 | 2% |
| 52 | Tolleston Dunes Trail | Indiana Dunes National Park | 18 | 62 | 80 | 1 | 0 | 1 | 1% |
| 53 | Leonard Springs Nature Park Loop | Bloomington, IN | 26 | 55 | 81 | 1 | 0 | 1 | 1% |
| 54 | Mason Ridge Trail | Morgan Monroe State Forest | 22 | 65 | 87 | 0 | 1 | 1 | 1% |
| 55 | Pokagon State Park Trail 3 | Pokagon State Park | 17 | 81 | 98 | 0 | 1 | 1 | 1% |
| 56 | Little Calumet River and Mnoke Prarie Trail | Indiana Dunes National Park | 20 | 79 | 99 | 0 | 1 | 1 | 1% |
| 57 | Charlestown State Park #2 Trail | Charlestown State Park | 28 | 84 | 112 | 0 | 1 | 1 | 1% |
| 58 | Tecumseh Trail | Morgan Monroe State Forest | 45 | 78 | 123 | 0 | 1 | 1 | 1% |
| 59 | Patton Cave via Grubb Ridge Loop | Charles C. Deam Wilderness | 15 | 124 | 139 | 1 | 0 | 1 | 1% |
| 60 | Cowles Bog Trail | Indiana Dunes National Park | 114 | 305 | 419 | 1 | 2 | 3 | 1% |
| 61 | Yellowwood Lake Trail | Yellowwood State Forest | 35 | 126 | 161 | 0 | 1 | 1 | 1% |
| 62 | Ogle Lake Trail 7 | Brown County State Park | 43 | 181 | 224 | 0 | 1 | 1 | 0% |
| 63 | Pate Hollow Trail | Hoosier National Forest | 42 | 197 | 239 | 1 | 0 | 1 | 0% |
| 64 | Camp Creek and Fall Creek Trails Loop | Fort Harrison State Park | 104 | 207 | 311 | 0 | 1 | 1 | 0% |
| 65 | Brown County State Park: Trail 8 | Brown County State Park | 61 | 250 | 311 | 0 | 1 | 1 | 0% |
